# Influence of irrigation conditions in the germination of plasma treated Nasturtium seeds

**DOI:** 10.1038/s41598-018-34801-0

**Published:** 2018-11-06

**Authors:** Ricardo Molina, Carmen López-Santos, Ana Gómez-Ramírez, Alberto Vílchez, Juan Pedro Espinós, Agustín R. González-Elipe

**Affiliations:** 1 Department of Biological Chemistry, Plasma Chemistry Group, Institute of Advanced Chemistry of Catalonia (IQAC), Consejo Superior de Investigaciones Científicas (CSIC), Jordi Girona 18-26, 08034 Barcelona, Spain; 2 Nanotechnology on Surfaces group, Institute of Materials Science of Seville (US-CSIC), Consejo Superior de Investigaciones Científicas, C/Américo Vespucio 49, 41092 Seville, Spain; 3 Atomic, Molecular and Nuclear Physics Department, University of Seville, Avda. Reina Mercedes, 41012 Seville, Spain

## Abstract

Plasma treatments had emerged as a useful technique to improve seed germination. In this work we investigate the influence of different irrigation conditions and plasma treatments on the germination of nasturtium seeds. During plasma treatment, seeds experience a progressive weight loss as a function of treatment time that has been associated to water release, a process that is more pronounced after longer plasma treatment times. Seeds treated for short times (<30 s) are able to germinate more efficiently than untreated specimen under hydric stress (drought conditions), while plasma treatments for longer times (up to 300 s) impaired germination independently on irrigation conditions. Characterization analysis of plasma treated seeds by FTIR-ATR, SEM/EDX and XPS showed that plasma treatment affected the chemical state of pericarp while, simultaneously, induced a considerable increase in the seeds water uptake capacity. The decrease in germination efficiency found after plasma treatment for long times, or for short times under optimum irrigation conditions, has been attributed to that the excess of water accumulated in the pericarp hampers the diffusion up to the embryo of other agents like oxygen which are deemed essential for germination.

## Introduction

Seed germination is a complex physiological process that begins with water uptake by the seed (imbibition) and ends with the emergence of the radicle^1^. Both the seed surface characteristics (e.g., specific morphology, structure, or composition) and its surrounding environment (e.g., moisture content, temperature, etc.) are known to influence the kinetics and amount of water uptake by the seeds. Availability of oxygen and its diffusion through the seed coat towards the radicle is another requisite for germination, particularly at the initial stages of embryonic cell division^2–4^. Water absorption into seed tissues can occur in different ways^5,6^. According to Bewley *et al*.^7^, the dormant seed cells of the embryo absorbs water at great rate, a process that induces their activation and division with the contribution of oxygen in creating energy transport vectors^2,8^. This behavior contrasts with that of other seed tissues that experience no expansion upon water uptake. Seed imbibition by water immersion, i.e. in the presence of an excess of water, can impair the embryo germination due to fast water absorption and slow diffusion of oxygen^9^. Different methods of modifying the seeds surface composition and other surface properties related with the seed-medium interaction, are currently used to improve seed germination, likely because of the modification of surface hydrophilicity and water uptake capacity^10^. Current procedures encompass methods that affect the integrity of the seed coat, such as treatments with concentrated sulphuric acid^11^, dry heat, hot water^12^ or NaOCl^13,14^. A more recent approach consists of the use of coatings made of superabsorbent polymers (e.g. polyacrylamide or acrylic acid)^15^ that absorb water from the surrounding soils, hold it at the seeds surface and contribute to increase germination capacity^16,17^.

Recently, the modification of surface properties of seeds by cold plasma treatments has emerged as an useful technique to improve seed germination^18–24^. Plasma active species (i.e. free radicals, excited atoms, molecules) are known to modify surface roughness and chemical composition of soft and organic materials in depths up to several tenths of nanometers^25^. On the other hand, vacuum ultraviolet radiation (VUV) and ultraviolet radiation (UV) emitted as a consequence of electronic transitions in the plasma discharge can induce photochemical reactions in polymers at deeper depths (i.e. breaking bonds, formation of free radicals, promote water desorption). In polymers, penetration depth for high energetic VUV photons (λ < 200 nm) is estimated in the range of 20 nm–100 nm, whereas penetration depth for UV photons can excess 1 µm^26–29^. In seeds, depending on the chemical composition of plasmas, similar changes are known to delay or, most commonly, accelerate seed germination^30^. In fact, germination behavior of plasma treated seeds is not univocal and, though in most cases a positive seed germination effect takes place^24,31–33^, in others germination rate remains unaffected or even decreases^21,34^.

Trying to shed some light into the complex processes involved in germination and its affectation by plasma treatments, in the present work we study the effect of atmospheric pressure plasmas on the germination of nasturtium seeds. The mature fruit of nasturtium is devoid of endosperm and breaks up into three single-seeded parts. Each seeded part is composed by an external pericarp and a thin testa that envelopes the thick and fleshy cotyledons and the embryo, this latter containing the reserve substances^35^. Pericarp in nasturtium seed has typical dimensions in the range of 1 mm, whereas the seed has a size in the order of 1 cm. During imbibition, rehydrated pericarp can act as a water reservoir and control the water and oxygen transportation to the cotyledons and embryo.

The purpose of this work is to investigate the interplay between plasma conditions and water content of the soil in inducing germination. For this purpose, we investigate the influence of different irrigation conditions and plasma treatments on the germination of nasturtium seeds. The basic hypothesis of this work is that water and oxygen diffusion properties through the rather thick pericarp of these seeds can be affected by the plasma treatments. Besides determining the optimal irrigation conditions, we show that only below a certain level (i.e., under hydric stress) plasma treated seeds germinated more efficiently than untreated specimen. The obtained decay/improvement in germination capacity upon plasma treatment has been correlated with the changes induced by plasma in the water uptake capacity and in the chemical state at the external seed coat as determined by infrared (FTIR), X-ray photoelectron (XPS) spectroscopy and X-ray emission spectroscopy in a SEM microscope.

## Results

### Germination rate of Nasturtium seed

**Figure 1:**
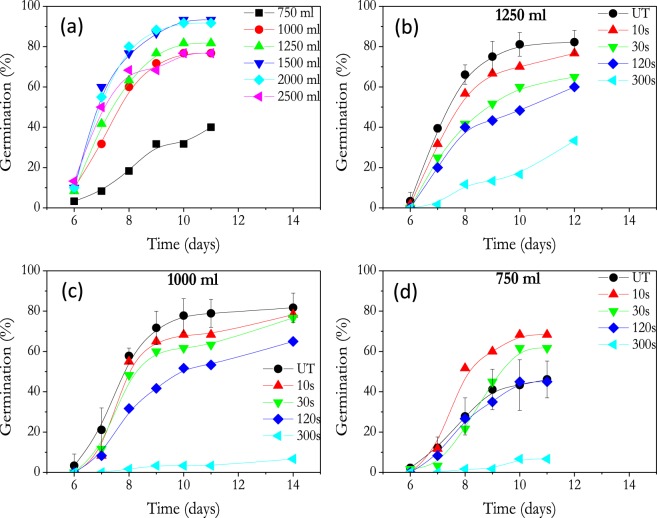
Germination rate of untreated (UT) and atmospheric plasma treated nasturtium seeds for the indicated periods of time. (**a**) Untreated nasturtium seeds as a function of irrigation conditions. (**b**–**d**) Germination for untreated and plasma treated nasturtium seeds for water irrigations with 1250 ml, 1000 ml and 750 ml, respectively.

The germination rate of untreated nasturtium seeds as a function of the amount of water used for irrigation is presented in Fig. 1a. Optimal conditions to achieve 90% energy germination (i.e., germination percentage after ten days)^36^ corresponded to 1500 ml and 2000 ml of water. A decrease in germination degree was found for irrigations with less and more water amounts than those of these optimal conditions (i.e., from 750 ml to 1250 ml and 2500 ml, respectively). It was particularly remarkable that for conditions close to hydric stress (i.e., 750 ml) germination efficiency drastically dropped.

Germination percentages for the nasturtium seeds previously treated with the atmospheric DBD plasma and exposed to different irrigation conditions are reported in Fig. 1b–d. In most experiments, with water irrigations either below or above optimal conditions (i.e., 1500–2000 ml), a decrease in germination was always observed for plasma treated samples (c.f., Fig. 1b,c). Independent of water irrigation conditions germination decreases as a function of plasma treatment time. It is particularly noteworthy the dramatic decrease in germination for seeds treated for long times (e.g., 300 s). However, for the lowest water irrigation conditions (i.e., 750 ml, Fig. 1d) the germination energy notably increases for short time plasma treated seeds (68.3 and 61.7% for 10 and 30 s of plasma treatment) and therefore, under these hydric stress irrigation conditions and short plasma treatment times, plasma treated seeds germinated more efficiently than untreated specimen.

### Weight losses induced by plasma and heat treatments

**Figure 2:**
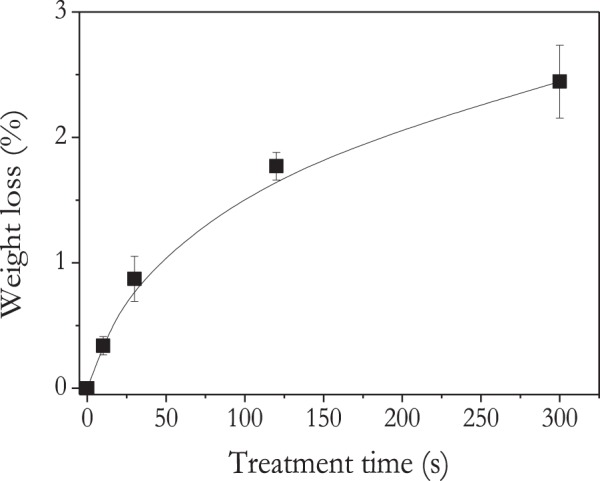
Nasturtium seed weight loss as a function of plasma treatment time.

Figure 2 shows that seeds experienced a net loss of weight during plasma treatment. For example, a noticeable 2.5% decrease in weight loss was observed after treatment for 300 s. Plasma etching processes should induce the complete oxidation of the outmost layers of a polymer substrate to CO_2_ and H_2_O^37^. It may also induce a certain desorption of water^38^. Weight loss directly due to the plasma etched outermost layers of seeds (i.e. maximum of a 1 μm after 300 s taking into account stronger DBD plasma conditions^39^) can be estimated in the order of 0.01% for a planar surface, i.e. it is negligible with respect to the determined 2.5% of weight loss and the actual dimensions of pericarp (≈1 mm) and nasturtium seed (≈1 cm). Therefore, we assume that weight losses resulting from plasma treatments are mainly due to water desorption.

A lateral effect of plasma treatments might be to increase the seeds temperature. Although a direct measurement of temperature in the plasma discharge zone during seed treatments is not possible because thermocouples or coloured strips would disrupt the plasma itself, measurements, just after 300 s plasma treatments yield values which never surpassed 35 °C. At this temperature, practically no weight losses could be detected after keeping the seeds at this temperature for one hour in an oven. However, weight losses could be induced thermally by heating at higher temperatures from 60 °C to 80 °C. These heating treatments for one hour also produced a certain degradation in the germination capacity of the seeds, as reported in Table 1. Data in this table show that germination of nasturtium seeds is not significantly affected at T < 70 °C, even if weight losses of the order of 2.8% were already found at 60 °C. A drastic decrease in germination was found for T > 70° and weight losses reaching up to 4.6% at 80 °C. However, seed temperature after heat treatments (even at 80 °C during 1 hour) decreases drastically in few seconds and the measured seed temperature (40 °C) is close to that found for plasma treated seeds (35 °C). Therefore, without discarding a contribution of thermal effects to the observed weight losses upon plasma treatments, we also assume that other non-thermal mechanisms of water desorption due to the plasma modification of the pericarp outer layers could take place (c.f., Fig. 2). These non-thermal mechanisms would be responsible for the difference observed between the times needed to observed the same water loss level either through a thermal treament (in the range of hours) or with plasma treament (minutes).

**Table 1:** Effect of temperature on weight loss and germination of nasturtium seeds.

Temperature (°C)	Weight loss after 1 hour (%)	Germination (%)
60	2.8	90.9%
70	3.8	84.5%
75	4.0	9.1%
80	4.6	0%

### Wetting properties and water uptake by plasma treated seeds

**Figure 3:**
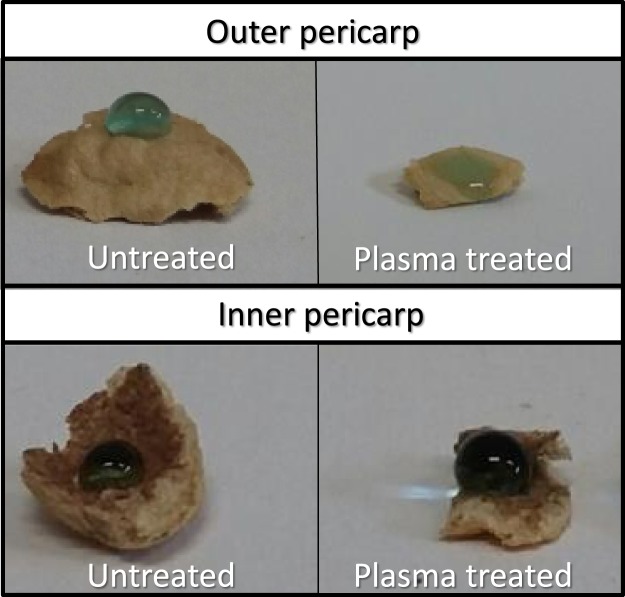
Water apparent contact angle of untreated and plasma treated (30 s) nasturtium seeds in the outer and inner part of the pericarp. A very diluted methylene blue concentration was used in order to better appreciate the water drop.

Surface of fresh nasturtium seeds are partially hydrophobic with water apparent contact angles close to 90°, but became highly hydrophilic (wetting apparent contact angles lower than 30°) after 30 s of plasma treatment (see Fig. 3). This behaviour is common for other plasma treated organic materials and has been related with their surface activation and the incorporation onto the surface of hydrophilic functional groups such as -OH, -COO-, etc^33^. Plasma activated seeds also respond to this general effect of plasma interaction with organic materials^40,41^. On the other hand, contact angle in the inner part of the pericarp (in contact with the thin testa that envelopes the thick and fleshy cotyledons and the embryo) does not seems to change after plasma treatment suggesting that the chemical modifications promoted by plasma active species and VUV/UV radiation are limited to the outer part of the pericarp and consequently the embryo and cotyledons would be unaffected by plasma treatment (althought thermal effects or other mechanism concerning water loss must not be disregarded).

However, contact angle is strongly influenced by the high roughness of nasturtium seed surface and consequently only qualitative information about modifications in hydrophobicity/hydrophilicity as a consequence of plasma treatment can be reported. Therefore the quantitative evaluation of the seed coat surface hydrophilicity promoted by plasma treatments was performed by means of the evaluation of water uptake and water vapor adsorption.

**Figure 4:**
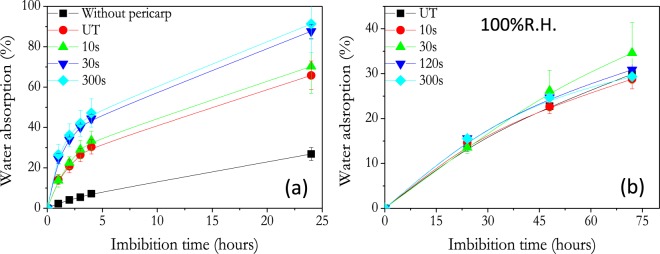
Weight increase of seeds due to water absorption (respect to the initial weigth) of nasturtium seeds imbiting at 21 °C. (**a**) Water uptake from water rich environment for plasma treated seeds as a function of time (imbibition time). (**b**) Ibidem from water vapor (100% R.H.).

Changes in hydrophicilicty after plasma treatment were accompanied by drastic variations in the way how seeds interact with the medium, particullarly regarding their water uptake capacity when put in contact with a water rich environment (see experimental section for details). Figure 4 shows that after four hours in contact with water rich environment (imbibition), the weight of the untreated seeds augmented by 30% and that a progressive enhancement in water uptake occurred for seeds plasma treated for longer times (e.g., 47% increase for the seeds treated for 300 s but only 33% after a plasma treatment for 10 s). It is thus remarkable that water uptake reached ca. 90% after 24 hours for the seeds treated for 300 s, while it only increased by 60% for the non-treated specimen. An additional point deserving consideration is the significant lower water uptake found for nasturtium seeds devoid of pericarp. According to the data in Fig. 4a, water uptake by these seeds only represented a 20% after 24 hours in water rich environment. A statistical measurement of the weight of nasturtium seeds with and without pericarp (>50 seeds), shows that pericarp represents ≈26% of the total weight of seeds. Therefore, the high amount of water uptake found for nasturtium seeds with pericarp must be mainly attributed to the swelling and rehydration of this latter which, in this way, would act as a water reservoir during germination (i.e., pericarp would act as a natural water absorbent polymer that envelops the testa and control water transportation to the embryo).

A different water uptake behaviour was found for experiments carried out in saturated water vapor (100% R.H.). Figure 4b reveals that in this case water uptake kinetic was much slower and the amount of water uptake smaller than from water rich environment and almost independent on plasma treatment time. Similar results have been observed with other seeds^33^. Gas exchange capability of seeds depends mainly on a combination of the structural arrangement of cells and intercellular spaces, and on the existence of distinct gas diffusion barriers^8^. Since water vapor adsorption it is almost independent on plasma treament time, it is suggested that under the plasma treatment conditions used in this work, the seed cell structure and porosity of nasturtium seeds it is not significantly modified.

### Chemical effects of plasma on the coat of nasturtium seeds

**Figure 5:**
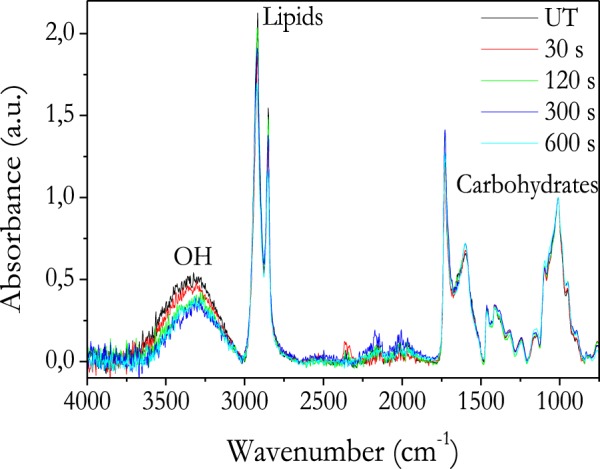
FTIR-ATR of the same nasturtium seed treated with plasma at different times.

The enhancement in the water uptake capacity of the seeds subjected to plasma treatments must be due to changes in the chemical state of their outmost surface layers. A first assessment of these changes was obtained by FTIR. In the ATR modality used in the present experiment the sample penetration depth is in the order of 1.5 µm^42^, much thinner than the thickness of the pericap of nasturtium seed (≈1 mm). This means that the possible changes monitored by this technique correspond to the outer layers of the seed coat without any contribution from the cotyledons. Since chemical composition of the pericarp might depend on the maturity degree of each particular nasturtium seed, FTIR analysis was performed with a unique seed specimen subjected to plasma treatment for increasing periods of time (Fig. 5).

Normalized FTIR-ATR spectra of nasturtium seeds in Fig. 5 are characterized by different peaks and bands that can be assigned to streching vibration in OH (3600–2990 cm^−1^), CH (2990–2744 cm^−1^) or C=O of carbonyl groups in lipids (1732 cm^−1^), aromatic stretching band of lignin (1602 cm^−1^), CH deformations and bending vibrations of OH (1473–1296 cm^−1^), C-O stretching of carbohydrates (cellulose and hemicellulose) (1246 cm^−1^), C-O deformation of carbohydrates and lignin (1187–857 cm^−1^)^43,44^. As indicated in Fig. 5, these latter bands have been linked with the presence of lipids and carbohydrates as constituent components of the seed coat^2^. On the other hand, the series of spectra in this figure measured after increasing plasma treatment times reveal no significant differences in shape, but a clear decrease in the band assigned to streching vibration in OH groups (3600–2990 cm^−1^). Since sample penetration depth of FTIR-ATR is in the order of 1.5 µm and similar to the penetration depth of VUV/UV radiation (very high compare to penetration depth of plasma active species (tens of nanometers)), this results suggest that not significant chemical reactions are promoted by VUV/UV radiation in the analysed depth. Therefore, since no additional new chemical groups are formed in the pericarp, it is suggested that the decrese in OH band intensity could be related to loss of water (weak bonded) from the pericarp outer zone and agrees with that the seed weight loss after plasma treatment (Fig. 2) is mainly due to water removal. Water removal in nasturtium seeds could take place as a consequence of water desorption promoted by VUV/UV radiation or local heating during plasma treatment. However, water loss could also take place in a greater depth than the FTIR-ATR penetration depth (≈1.5 µm).

**Figure 6:**
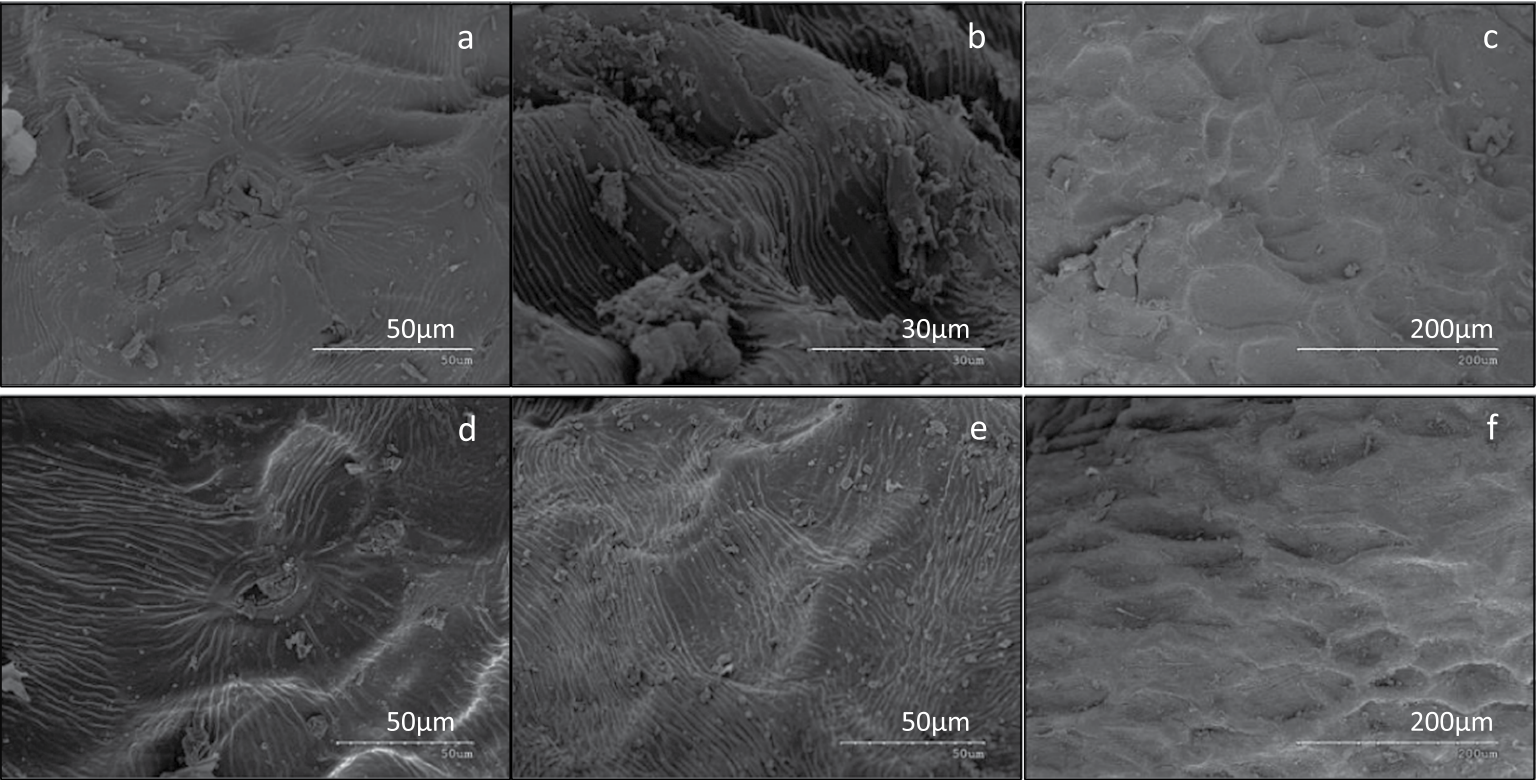
SEM surface images at different magnifications corresponding to untreated (up) and plasma treated nasturtium seeds (down) for 300 s.

**Figure 7:**
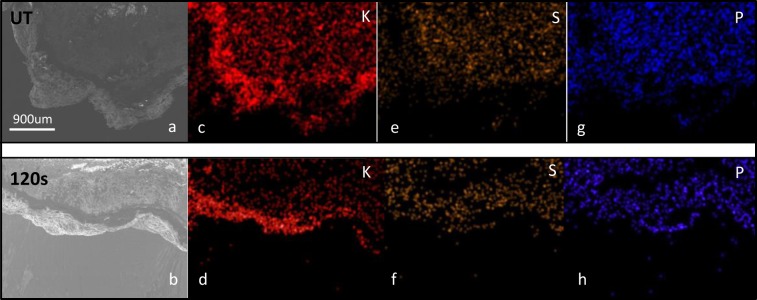
(**a**,**b**) Low magnification SEM micrographs of seed cuts before and after plasma exposure for 120 s. (**c**–**g**) EDX maps of the distribution of K, S and P before and after plasma treatments.

A cross section analysis by SEM-EDX of the samples before and after plasma treatement complements these results providing information about the composition in a thickness in the order of microns. The outer surface morphology of the nasturtium seed did not seem to be modified by the plasma treatment, at least by SEM observation and for the treatment times used in this work (see Fig. 6). Different authors evidenced by SEM analysis that plasma treatment can induced significant changes on the seeds’ surface, which was related to water permeability into the seeds^40,41^. However, our SEM and water vapor adsorption (Fig. 4b) results suggest that the porosity of nasturtium seeds has not ben modified by the plasma conditions used in this work and consequently it is suggested that the water uptake increase observed (Fig. 4a) could be related with the increase in surface hydrophilic chemical groups promoted by the plasma treatment. The cross section EDX analysis of the distribution of minority components such as K, S and P reveals that some modifications were induced, at least indirectly, by the plasma treatments. Figure 7 shows EDX mapping of these elements on seed cuts extending from the surface up to some microns inside the pericarp. From the intensity of the color maps it is apparent that while K becomes enriched in the most outer layers of the coat within a thickness of approximately 300 µm, the distribution of the other investigated elements became either unaffected or their concentration slightly depleted. In previous works, a considerable diffusion of potasium towards the surface in quinoa seeds and post-oxidation changes in pepper seeds after plama treatment were reported^33,45^.

Additional information about composition and chemical state of elements in a much shallower sample depth can be obtained by XPS analysis (average depth of analysis of approximately 2 nm)^46^. A survey spectrum of the seeds reveals the presence of carbon, oygen, nitrogen and some potassium at the surface. From the intensity of these peaks it is possible to determine surface chemical composition in atom percents. The obtained data, gathered in Table 2, reveal a majority partition of carbon atoms, as expected from a seed coat mainly composed of lipids and carbohydrates. Additonally oxygen, nitrogen and potassium were also detected as constituent elements of the coat surface. Plasma treatments for short period of times (10 and 30 s) lead to a relative decrease in carbon and to increases in oxygen, nitrogen and potassium. After longer treatment times, from 30 s to 300 s, no significant variation in the surface elemental composition was detected. This kind of steady state conditions suggest that after 30 s of plasma treatment, full oxidation of the outermost surface is taking place, leading to the release of CO_2_ (and H_2_O) formed as a consequence of the complete oxidation of carboxylated groups detected after 30 s plasma treatments and that, in this way, would be the intermediate species in these plasma etching removal processes. Similar processes has been observed in plasma treated wool, where surface chemical composition reached a steady state after 40 s of plasma treatment time^37^.

**Table 2:** Elemental surface composition in atom percentages for plasma treated nasturtium seeds.

	%C	%O	%N	%K
Untreated	86.4	12.1	1.2	0.3
10 s	73.8	23.1	2.1	1.0
30 s	73.1	24.2	1.5	1.2
120 s	70.8	25.9	2.2	1.1
300 s	70.6	25.7	2.4	1.3

**Figure 8:**
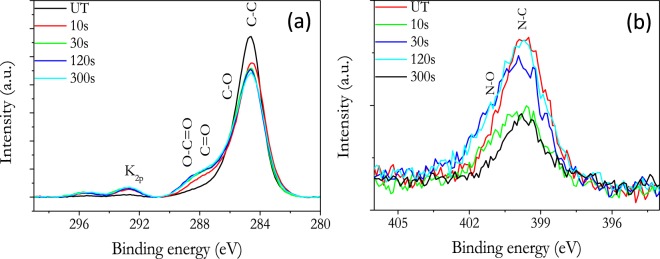
XPS high resolution C_1s_/K_2p_ (**a**) and N_1s_ (**b**) spectra corresponding to untreated and plasma treated nasturtium seeds.

High resolution spectra corresponding to carbon and potassium (C_1s_/K_2p_ peaks) confirmed these trends (Fig. 8a). For short plasma treatment times (10 s and 30 s) an increase in the carbonyl (C=O, 288.3 eV) and carboxyl (O-C=O, 289.4 eV) hydrophilic groups took place, while no significant variation in the carbon functionalities was found after 30 s of plasma treatment. The reported surface transformation from partially hydrophobic to highly hydrophilic after plasma treatments is likely related with the formation of these hydrophiclic groups at the surface. On the other hand, the high-resolution N_1s_ spectra in Fig. 8b shows a clear increase in the band at ≈399.8 eV with respect to the untreated sample. This rather broad band (i.e., around 3 eV of FWMH) can be attributed to the convolution of different C-N bonding groups (e.g, C-NH_2_, OC-NH_2_, C-N, etc.)^47^ which, having contributions relatively close in BE, cannot be resolved in the spectrum. After prolonged plasma exposure, the shape of the convolution band developed a little shoulder in the region (400–402 eV) attributed to nitrogen oxides. The origin of these surface C-NO_x_ groups must be linked to the oxidation of C-N surface groups, although the adsorption of NO_x_ species formed in the plasma phase cannot be discarded^48^. Overall, the formation of these surface groups can be interpreted as resulting from a progressive plasma oxidation removal of the lipids and carbohydrates forming the surface coat. This oxidation occurs thorugh the formation of oxidative surface groups (C-OH, CO, COOH/COO^−^) which are responsible for the hydrophilic transformation of the surface state and that, by further oxidation, must lead to the formation of CO_2_ (and H_2_O) and the progressive etching of the seed coat.

It is noteworthy in this analysis that, in agreement with the EDX maps in Fig. 7, the XPS spectrum shows an enrichment of potassium after plasma treatment, a result that coincides with a similar observation on quinoa seeds subjected to the effect of oxidative plasmas^33^. We tentatively propose that this out diffusion of potassium is a result of electrostatic interactions with negatively charged surface groups formed during plasma treatment (e.g., -COO^−^, -NO_x_^−^). Although finding the implications of a seed surface layer with net positive and negative charges in the control of water diffusion would deserve more specific experiments, it is a likely hypothesis that this outer surface layers strongly interact with water favouring their diffusion to the interior of the pericarp.

Taking into account that seed germination decreases (c.f. Fig. 1) and water absorption capacity increases (c.f. Fig. 4) for plasma treatment times longer than 30 s, we assume that, at least for nasturtium seeds characterized for a rather thick pericarp, the nature of the new surface chemical groups formed at the surface by plasma treatment are not critical in directly determining the decrease in germination found for seeds treated for long plasma treatment times, although they might play a signifinct role in water diffusion mechanisms. However, longer plasma treatment times should promote additional transformations in the pericarp outer layers which, not detected in the present work, would be responsible for the observed enhancement in water absorption. We tentatively propose that the parallel decrease in germination energy results from an excess of water incorporated into the pericarp^49^.

## Discussion

The previous results have evidenced a series of key features regarding the effect of plasma and irrigation conditions on the germination of nasturtium seeds. They can be summarized in the following points:

- Plasmas at short times enhance the germination energy under hydric stress conditions (c.f. Fig. 1).
- Water uptake is greatly enhanced by plasma treatment (c.f., Fig. 4).
- Very short plasma treatment times are sufficient to achieve a steady state functionalization of the seed surface, while longer plasma treatment times lead to progressive weight losses, an excess of water uptake and a decrease in germination energy (c.f., Figs 1, 2, 4, 8).

A first analysis of these findings is that plasma treatment alter the membrane properties of the pericarp increasing water uptake processes. On the other hand, the water uptake experiments with the seeds devoided from pericarp (c.f. Fig. 4) suggests that the majority of water incorproated in the seed during the first 24 hours in contact with a water rich environment is located in the pericarp which, in this way must be fully saturated with water after that time. This saturation effect was enhanced in seeds plasma treated for longer times. Besides water, oxygen is essential to promote germination through its participation in the chemical synthesis of energy vectors (i.e., mitochondrial ATP) in the embryo cells^8^. Although the diffusion of oxygen from the medium towards the embryo under humid conditions has been modelled for some seeds^4^, there are not systematic studies on the influence of humidity on such processes. Diffusion rate of oxygen in water is order of magnitudes smaller than in air or through organic tissues^50,51^ and, in the nasturtium seeds with a very thick pericarp oxygen flow up to the embryo might be drastically reduced in excess of water. We propose that an excessive incorporation of water in the pericarp impairs the oxygen transportation to the interior of seeds and, therefore, produces a decrease in the seed germination capacity (a slow or hampered oxygen diffusion through the pericarp will prevent a vigorous respiration and other oxidative processes involved in germination of most species)^9,52,53^.

The pervasive effect of plasma in decreasing the germination capacity of nasturtium seeds (except for the lowest irrigation conditions, c.f. Fig. 1d) contrasts with the generally positive effect of similar treatments caused on other seeds^24,31–33^. In the case of nasturtium seeds, the rather thick pericarp seems to be a differential feature significantly affecting the oxygen and water diffusion to the cotyledons and embryo. The preferential location of absorbed water in the pericarp deduced from the experiments in Fig. 4 suggest that during water uptake pericarp swells and acts as a water reservoir for germination. i.e., pericarp behaves as a natural hydro-absorbent polymer that envelopes the testa and modifies water and oxygen transportation to the embryo. In this regard, the effect of plasma treatment seems very similar to the effect hydro-absorbent polymer coatings on seeds^16,17^. In particular, germination becomes reduced when excess of water uptake by the pericarp restricts embryonic oxygen availability^54^. Under this scheme, it is natural the positive effect of plasma on germination found when the availability of water supply is scarce (Fig. 1d). Under these conditions, even if plasma favors the water uptake, this does not saturates the pericarp because its little availability in the medium, and the oxygen diffusion to the embryo does not become restricted.

The chemical analysis data by FTIR, XPS and EDX-SEM provide some clues to understand why plasmas contribute to enhance the water diffusion through the pericarp surface (i.e., it transforms the pericarp from hydrophobic to hydrophilic). XPS results in Fig. 8, shows that surface reaches steady state composition after 30 s, while the IR bands associated to streching vibration in OH groups (3600–2990 cm^−1^) continuously decreases in intensity for longer treatment times. This difference evolution must be link to the different depths sampled by each technique (i.e., XPS get information from just 2 nm of surface thickness, while FTIR analyses a thickness of approximately 1.5 µm). The progressive decrease in the band associated to streching vibration in OH groups (3600–2990 cm^−1^) correlates with the weight losses measured by the plasma treated specimens that have been attributed to the loss of water from the pericarp. On the other hand, SEM and water vapor adsorption analysis suggest that the porosity of nasturtium seeds has not ben modified by the plasma conditions used in this work. Therefore, it seems that after short plasma treatment times (<30 s) changes in outer surface composition make that water diffusion trough the pericarp (and eventually up to cotyledons and embryo) is enhanced with respect to untreated nasturtium (Fig. 1d). In the particular case of this seed, the preferential incorporation of water in the pericarp may result deleterious if an excessive incorporation of water due to a prolonged plasma treatment impairs the diffusion of oxygen up to the embryo. The increase in germination rate under drought conditions for seeds plasma treated for 30 s supports that plasma treatments can be used as an alternative to hydro-absorbent polymer coatings on specific seeds.

## Conclusions

Germination of atmospheric plasma treated nasturtium seeds has been investigated as a function of irrigation conditions. It has been found that atmospheric plasma treatment contributes to increases water uptake of nasturtium seeds and that plasma treatments for short period of times (10 and 30 s) are enough to promote water uptake even under hydric stress (drought conditions) when it was beneficial for seed germination. However, at high irrigation conditions water uptake increase results in a decline in germination. It has been proposed that an excessive increase of water uptake in seeds with a very thick pericarp may impair the diffusion of oxygen towards the interior of the seed preventing its germination. It can be concluded that the use of plasma to favor germination is not a universal phenomenon and that a careful tradeoff analysis must be carried out in order to correlate irrigation conditions and plasma treatment time for each particular type of seeds.

## Methods

### Materials and germination conditions

Nasturtium seeds (Tropaeolum majus) were obtained from Semillas Fitó (Spain), using specimen freshly removed from hermetic packaging. Germination of plasma treated seeds was systematically compared with that of untreated seeds sowed and grown under equivalent irrigation conditions. Both control and plasma treated seeds were sowed (4 cm below the surface) in module trays (66 cells with a volume of 80 ml per cell) containing soil substrate (≈50 gr). For germination experiments, 3 replicas of 66 seeds for untreated seeds and 1 experiment with 132 seeds for each plasma treatment for the different irrigation conditions were performed. The soil substrate used (substrato semilleros, terreau semis) was obtained from Flower S.A. (Tarrega, Spain) and contained sphagnum peat moss, litonite, perlite, quartz crystals and fertilizer.

Water irrigation was done by capillarity uptake after seed sowing. At first day of seed sowing, one corresponding volume of water varying from 750 ml to 2500 ml was put inside a plastic tray (35 cm L × 50 cm W × 8 cm H) and the module tray containing the sowed seeds was put over the water. No more water was added during germination experiments. Soil moisture obtained after irrigation was therefore varied between 41.5% and 70.3%. Average ambient temperature during seed germination was ≈22 °C. The day when the plant raises from the soil substrate was taken as germination time of this specific specimen. Germination percentage was calculated using the equation:

Gemination (%) = 100 × (Number of germinated seeds)/(total number of seeds planted)

Indeed, germination percentage after ten days is defined as the germination energy (ISTA 2012)^36^.

### Atmospheric cold plasma treatment

**Figure 9:**
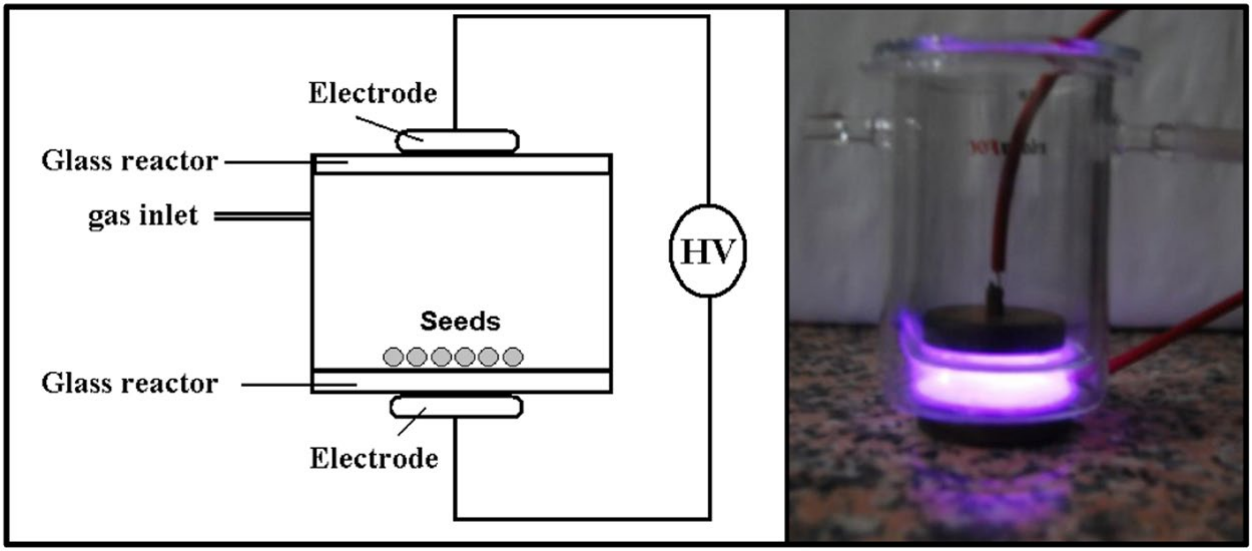
Experimental set-up for atmospheric plasma treatment of nasturtium seeds.

The atmospheric plasma used for the experiments was generated in a dielectric barrier discharge reactor (DBD)^55^, made of very simple glass containers and spacers (see Fig. 9). It consisted of two parallel metal electrodes (45 mm in diameter) separated by 12 mm and covered by glass acting as a dielectric plate. To avoid arching or microdischarges that might damage the seed surface coat, Helium was used as primary plasma gas. Its flow (5 L_n_ min^−1^) was controlled with a mass flow meter and controller (Bronkhorst, Ruurlo, Netherlands). Since the reactor had no tight closures (see the scheme in Fig. 1) air mixed with the Helium during experiments making that oxygen and nitrogen active species are in contact with the seeds^55^. A 16 kHz signal was generated with a GF-855 function generator (Promax, L’Hospitalet de Llobregat, Spain) connected to a linear amplifier AG-1012 (T&C Power Conversion, Inc., Rochester, NY, USA). A matching network and two transformers (HR-Diemen S.A., Sant Hipòlit de Voltregà, Spain) were connected to the amplifier output in order to increase the voltage up to 20 kV. Nasturtium seeds where placed onto the bottom glass covering the electrode, the incident power adjusted to 30 W and the exposure time changed from 10 s to 300 s.

### Characterization of seeds

Weight loss (%) induced by plasma treatment was determined weighting the seeds before and just after plasma treatment. Different replicas (at least 3) with at least 11 seeds per treatment were analyzed in order to get average weight losses.

1 $${{\rm{W}}}_{{\rm{loss}}}( \% )=100\times ({{\rm{W}}}_{i}-{{\rm{W}}}_{f})/{{\rm{W}}}_{i}$$

Liquid water uptake kinetics was carried out at 21 °C with destilled water in an impregnated Spandex scouring pad (water rich environment) for increasing periods of time up to 24 hours. Four replicas of the experiment with groups of 15 seeds per treatment were carried out. Water uptake experiments were also carried out exposing the seeds to humid air (100% humidity) in a close environment for increasing periods of time. By this experiment, seeds were placed over an open petri dish inside a desiccator with distilled water in the low compartment in order to achieve a relative humidity (RH) of 100%. RH inside the desiccator was controlled with a hygrometer (Diligence EV N2013, Comark limited UK).

Wetting behaviour of the seeds before and after plasma treatment was characterized by imaging observation of a small droplet of water (about 10 microliter) dripped onto the seed surface (note that the rough and curve character of the seed surface precludes an accurate determination of wetting contact angle). A 0.5 µM methylene blue (Certified by the Biological Stain Commission, Sigma-Aldrich) solution in water was used in order to better appreciate the water drop in Fig. 3. Surface tension of methylene blue solutions in the micromolar level (≈71.0 mN/m) is close to that of desionized water (≈72.0 mN/m)^56^.

FTIR-ATR analysis of the samples was carried out in a Nicolet AVATAR 360 spectrometer in the range of 400–4000 cm^−1^. Measurements were performed using the Smart iTR Attenuated Total Reflectance (ATR) Sampling Accessory (Thermo Scientific Inc., U.S.A). Spectra were obtained with an average of 32 scans using a resolution of 4 cm^−1^. An advanced ATR correction algorithm (OMNIC 7.3 from Thermo Electron Corporation) was used to correct for band intensity distortion, peak shifts and polarisation effects. Corrected ATR spectra were found quite comparable to their transmission equivalents^48^. Spectra were normalized to the peak corresponding to C-O deformation of carbohydrates and lignin (1010 cm^−1^).

The morphology of untreated and plasma treated seeds was assessed by scanning electron microscopy (model Hitachi S-4800) with an acceleration voltage of 5 kV. EDX elemental mapping of the interior of the seeds before and after plasma treatment was done with a Bruker-X Flash-4010 system after cutting and separating the seeds in two parts for analysis. In all cases no metal coating was applied during sample preparation.

X-ray photoelectron spectra (XPS) of treated and untreated seeds were acquired in a PHOIBOS 100DLD spectrometer working in the pass energy constant mode with the MgKα radiation as excitation source. Binding energy scale of the spectra was calibrated with the C_1s_ main peak at a value of 284.5 eV. For this analysis various seeds were covering the whole analysis area (0.5 × 0.5 cm) in order to avoid any contribution from the substrate.

## References

[1] Rajjou L (2012). Seed Germination and Vigor. Annu. Rev. Plant Biol..

[2] Bewley, J. D., Bradford, K. J., Hilhorst, H. W. M. & Nonogaki, H. *Seeds: Physiology of development, germination and dormancy*, 3rd edition 9781461446934 (Springer, 2013).

[3] Vertucci CW, Leopold AC (1984). Bound Water in Soybean Seed and Its Relation to Respiration and Imbibitional Damage. Plant Physiol..

[4] Budko, N. *et al*. *Oxygen transport and consumption In germinating seeds* (ed. Heydenreich, M.O. *et al*.) 5–30 (Proceedings of the 90th European Study Group Mathematics in Industry, 2013).

[5] Rodrigues AG, Oliveira TGS, Souza PPd, Ribeiro LM (2013). Water uptake and pre-germination treatments in macaw palm (Acrocomia aculeata - Arecaceae) seeds. J. Seed Sci..

[6] McDonald MBJ, Vertucci CW, Roos EE (1988). Soybean seed imbibition: water absorption by seed parts. Crop. science.

[7] Bewley, J. D. & Black, M. *Seeds: physiology of development and germination*. (Plenum Publishing Corporation, 1994).

[8] Borisjuk L, Rolletschek H (2009). The oxygen status of the developing seed. New Phytol..

[9] Marcos Filho, J. M. F. *Fisiologia de sementes de plantas cultivadas*. (Fealq, 2005).

[10] Kim SY, D. E Data SK, Mercado BL (1990). The effect of chemical and heat treatments on germination of Commelina benghalensis L. aerial seeds. Weed Res..

[11] Potter RL, Joseph LP, Ueckert DN (1984). Germination Responses of Opuntia spp. to Temperature, Scarification, and Other Seed Treatments. Weed Sci..

[12] Walker SR, Evenson JP (1985). Biology of Commelina benghalensis L. in south‐eastern Queensland. 1. Growth, development and seed production. Weed Res..

[13] Hsiao AI, Quick WA (1984). Actions of sodium hypochlorite and hydrogen peroxide on seed dormancy and germination of wild oats, Avena fatua L. Weed Res..

[14] Huang WZ, Hsiao AI (1987). Factors affecting seed dormancy and germination of Johnsongrass, Sorghum halepense (L.) Pers. Weed Research.

[15] Ekebafe L, Ogbeifun DE, Okieimen FE (2011). Polymer Applications in Agriculture. Biokemistri.

[16] Akelah, A. *Functionalized Polymeric Materials in Agriculture and the Food Industry*. (Springer, 2013).

[17] Hotta M, Kennedy J, Higginbotham C, Morris N (2016). Durum wheat seed germination response to hydrogel coatings and moisture under drought stress. AJABS.

[18] Masafumi I, Jun‐Seok O, Takayuki O, Masaharu S, Masaru H (2018). Current status and future prospects of agricultural applications using atmospheric‐pressure plasma technologies. Plasma Process. Polym..

[19] Dobrin D, Magureanu M, Mandache NB, Ionita M-D (2015). The effect of non-thermal plasma treatment on wheat germination and early growth. Innov. food Sci Emerg. Technol..

[20] Sera B, Gajdová I, Sery M, Špatenka P (2013). New Physicochemical Treatment Method of Poppy Seeds for Agriculture and Food Industries. Plasma Sci. Technol..

[21] Sera B, Sery M, Gavril B, Gajdova I (2017). Seed Germination and Early Growth Responses to Seed Pre-treatment by Non-thermal Plasma in Hemp Cultivars (Cannabis sativa L.). Plasma Chem. Plasma P..

[22] Puač N, Gherardi M, Shiratani M (2018). Plasma agriculture: A rapidly emerging field. Plasma Process. Polym..

[23] Măgureanu M, Sîrbu R, Dobrin D, Gîdea M (2018). Stimulation of the Germination and Early Growth of Tomato Seeds by Non-thermal Plasma. Plasma Chem.Plasma P..

[24] Sivachandiran L, Khacef A (2017). Enhanced seed germination and plant growth by atmospheric pressure cold air plasma: combined effect of seed and water treatment. RSC Advances.

[25] Bismarck, A. & Springer, J. *Wettability of Materials: Plasma Treatment Effects Encyclopedia of Surface and Colloid Science*. 6592–6610 (Taylor & Francis, 2006).

[26] Wertheimer MR, Fozza AC, Holländer A (1999). Industrial processing of polymers by low-pressure plasmas: the role of VUV radiation. Nucl. Instrum. Methods Phys. Res. B.

[27] Fridman, A. *Plasma Chemistry*. (Cambridge University Press, 2008)

[28] Raupp GB, Junio CT (1993). Photocatalytic oxidation of oxygenated air toxics. Appl. Surf. Sci..

[29] Misra DN (1972). Dehydroxylation of Anatase Surface by Irradiation and Nature of Adsorbed Water. Nature Physical Science.

[30] Volin JC, Denes FS, Young RA, Park SMT (2000). Modification of Seed Germination Performance through Cold Plasma Chemistry Technology. Crop Sci..

[31] Alves Junior C, Vitoriano JO, Layza Souza da Silva D, Farias M, Bandeira de Lima Dantas N (2016). Water uptake mechanism and germination of Erythrina velutina seeds treated with atmospheric plasma. Sci Rep..

[32] Bormashenko E, Grynyov R, Bormashenko Y, Drori E (2012). Cold Radiofrequency Plasma Treatment Modifies Wettability and Germination Speed of PlantSeeds. Sci. Rep..

[33] Gómez-Ramírez A (2017). Surface chemistry and germination improvement of Quinoa seeds subjected to plasma activation. Sci. Rep..

[34] Bormashenko E (2015). Interaction of cold radiofrequency plasma with seeds of beans (Phaseolus vulgaris). J. Exp. Bot..

[35] Hoth A, Blaschek W, Franz G (1986). Xyloglucan (amyloid) formation in the cotyledons of Tropaeolum majus L. seeds. Plant Cell Rep..

[36] ISTA. International Rules for Seed Testing. ISTA (2012).

[37] Molina R, Espinós J, Yubero F, Erra P, Gonzalez-Elipe A (2005). XPS analysis of down stream plasma treated wool: Influence of the nature of the gas on the surface modification of wool. Appl. Surf. Sci..

[38] Yamamoto T, Tanioka G, Okubo M, Kuroki T (2007). Water vapor desorption and adsorbent regeneration for air conditioning unit using pulsed corona plasma. J Electrostat..

[39] Dimitrakellis P, Gogolides E (2018). Atmospheric plasma etching of polymers: A palette of applications in cleaning/ashing, pattern formation, nanotexturing and superhydrophobic surface fabrication. Microelectron. Eng..

[40] Stolárik T (2015). Effect of Low-Temperature Plasma on the Structure of Seeds, Growth and Metabolism of Endogenous Phytohormones in Pea (Pisum sativum L.). Plasma Chem. Plasma P..

[41] Pawłat J (2018). Effects of atmospheric pressure plasma jet operating with DBD on Lavatera thuringiaca L. seeds’ germination. PLoS One.

[42] Averett LA, Griffiths PR, Nishikida K (2008). Effective Path Length in Attenuated Total Reflection Spectroscopy. Anal. Chem..

[43] Yu P (2003). Chemical Imaging of Microstructures of Plant Tissues within Cellular Dimension Using Synchrotron Infrared Microspectroscopy. J. Agric. Food Chem..

[44] Lisperguer J, Perez P, Urizar S (2009). Structure and thermal properties of lignins: characterization by infrared spectroscopy and differential scanning calorimetry. J. Chil. Chem. Soc..

[45] Vlasta Š (2018). Atmospheric pressure plasma treatment of agricultural seeds of cucumber (Cucumis sativus L.) and pepper (Capsicum annuum L.) with effect on reduction of diseases and germination improvement. Plasma Process. Polym..

[46] López-Santos MC, Yubero F, Espinós JP, González-Elipe AR (2010). Non-destructive depth compositional profiles by XPS peak-shape analysis. Anal. Bioanal. Chem..

[47] Beamson G, Briggs D (1993). High Resolution XPS of Organic Polymers: The Scienta ESCA300 Database. J. Chem. Educ..

[48] Molina R (2013). Surface Functionalization of Macroporous Polymeric Materials by Treatment with Air Low Temperature Plasma. J. Nanosci. Nanotechnol..

[49] Salis A, Monduzzi M (2016). Not only pH. Specific buffer effects in biological systems. Curr. Opin. Colloid Interface Sci..

[50] Wilke CR, Chang P (1955). Correlation of diffusion coefficients in dilute solutions. AIChE Journal.

[51] Massman WJ (1998). A review of the molecular diffusivities of H_2_O, CO_2_, CH_4_, CO, O_3_, SO_2_, NH_3_, N_2_O, NO, and NO_2_ in air, O_2_ and N_2_ near STP. Atmos. Environ..

[52] de Melo RB, Franco AC, Silva CO, Piedade MTF, Ferreira CS (2015). Seed germination and seedling development in response to submergence in tree species of the Central Amazonian floodplains. AoB PLANTS.

[53] Kozłowski, T. T. & Pallardy, S. G. *Growth control in woody plants*. (Academic Press, Inc., 1997).

[54] Gorim L, Asch F (2017). Seed Coating Increases Seed Moisture Uptake and Restricts Embryonic Oxygen Availability in Germinating Cereal Seeds. Biology.

[55] Molina R, Ligero C, Jovančić P, Bertran E (2013). *In Situ* Polymerization of Aqueous Solutions of NIPAAm Initiated by Atmospheric Plasma Treatment. Plasma Process.Polym..

[56] Ardizzone S, Gabrielli G, Lazzari P (1993). Adsorption of Methylene Blue at solid/liquid and water/air interfaces. Colloids Surf. A..

